# Editorial: Communication of Risk in the Public Realm

**DOI:** 10.3389/fpsyg.2022.935352

**Published:** 2022-07-11

**Authors:** Manuele Reani, Caroline Jay, Alvitta Ottley

**Affiliations:** ^1^School of Management and Economics, The Chinese University of Hong Kong, Shenzhen, China; ^2^Department of Computer Science, The University of Manchester, Manchester, United Kingdom; ^3^Department of Computer Science and Engineering, McKelvey School of Engineering, Washington University in St. Louis, St. Louis, MO, United States

**Keywords:** risk, biases, decision making, conspiracy theories, media communication, misinformation

It is well-documented that people struggle to understand risk, an issue that has recently been evident in both the public and governments' responses to the COVID-19 pandemic (Montagni et al., 2021). This Research Topic sought to examine why and how people fail to understand risk and uncover interventions for improving risk communication to the public.

Under the COVID-19 pandemic, optimizing how governments and institutions communicate health information is paramount. Specifically, risk communication has been challenging because most people tend to struggle with statistical reasoning (Hoffrage et al., 2000). Therefore, the communication of risks involves presenting statistical information, often using specific visualization techniques (Ottley et al., 2015; Reani et al., 2018, 2019a,b). A specific technique called infographics, for instance, has been introduced for this purpose which seems to alleviate some of the issues inherent in understanding probabilities (Spiegelhalter et al., 2011; Mosca et al., 2021). Still, technological advances have shifted the mode of communication to web spaces, introducing additional opportunities and challenges, including misinformation and disinformation (Lee et al., 2021).

Early research in behavioral decision-making has shown that people's cognitive abilities are limited: they often fall victim to biases and use heuristics to make decisions, even when their health is at risk (Kahneman, 2011). The review article by Edwards addresses this issue by presenting the journey from (1) behavioral economic theories to (2) heuristics research to (3) behavioral analysis, concluding with a discussion on how to best present public health information in a way that minimizes human biases. This research has practical implications as it lays the foundation for understanding the change in paradigms that governments and institutions need to make if they want to communicate effectively with the public, especially in light of the recent events related to the COVID-19 pandemic.

Two related phenomena that have increased in recent years are the generation and dissemination of fake news, especially on the Web, and the development of conspiracy theories (Oleksy et al., 2021). It is often hard to determine the origin of conspiracy theories and the effect that such opinions might have on people's beliefs and behaviors. It is even more challenging to find a solution to this problem. The article by Leonard and Philippe examines the significant increase in the endorsement of conspiracy theories related to the SARS-CoV-2 pandemic. The authors present a narrative review exploring why conspiracy theories related to healthcare topics emerge. According to their discussion, mistrust of the authorities seems to be one of the major culprits. To mitigate this phenomenon, they suggest that governments and organizations will need to increase citizen engagement to build trust and propose initiatives to support this. This research is necessary if we want to bring risk communication to the next level.

A further article examines the issue of social integration and emotional wellbeing in virtual communities on the Web. Zhang et al. highlight that virtual communities are becoming more critical, especially for younger generations, since the onset of the COVID-19 pandemic. The pandemic brings risk and uncertainty about the future of society, and people explore this online. Thus, understanding how virtual communities behave is vital. The article focuses on the importance of supporting healthy interactions on the web. It highlights that self-disclosure is a crucial determinant of psychological wellbeing that can boost social integration levels in online communities. As our lives move toward cyberspace, we need to ensure it becomes a more positive and healthier place.

Choudhary and Dut propose a solution to one of the most pressing problems: climate change. They tackle the issue of people preferring to take a “wait-and-see” approach over early intervention through the Interactive Climate Change Simulator (ICCS). This Web-based tool enables people to simulate the impact of investment in climate change mitigation and obtain feedback on the results of different actions. They demonstrate that the ICCS tool helped alleviate people's tendency to “wait-and-see” and increased their potential investments to counteract climate change. Simulation tools like ICCS have the potential to improve people's understanding of climatic disasters and can act as a helpful aid for educationalists and policymakers.

Altogether, the collection of articles highlights critical challenges in risk communication, addressing current, real-world topics such as communication for online spaces, COVID-19, and climate change. We are grateful to all the contributors to this Research Topic and hope that they catalyze further innovations.

## Author Contributions

MR wrote the initial draft of the editorial. CJ contributed to writing the editorial. AO contributed to writing the editorial and revised the final version. All authors contributed to the article and approved the submitted version.

## Funding

This material is based upon work supported by the National Science Foundation under grant number 1755734.

## Conflict of Interest

The authors declare that the research was conducted in the absence of any commercial or financial relationships that could be construed as a potential conflict of interest.

## Publisher's Note

All claims expressed in this article are solely those of the authors and do not necessarily represent those of their affiliated organizations, or those of the publisher, the editors and the reviewers. Any product that may be evaluated in this article, or claim that may be made by its manufacturer, is not guaranteed or endorsed by the publisher.
